# Comparison of stool samples and rectal swabs with and without pre-enrichment for the detection of third-generation cephalosporin-resistant Enterobacterales (3GCREB)

**DOI:** 10.1007/s10096-021-04250-1

**Published:** 2021-04-27

**Authors:** Tarek Jazmati, Axel Hamprecht, Nathalie Jazmati

**Affiliations:** 1 Institute for Medical Microbiology, Immunology and Hygiene, University of Cologne, Goldenfelsstrasse 19-21, 50935 Cologne, Germany; 2 German Centre for Infection Research (DZIF), partner site Bonn-Cologne, Cologne, Germany; 3 Institute for Medical Microbiology and Virology, University of Oldenburg & Klinikum Oldenburg, Oldenburg, Germany; 4 Laboratory Dr. Wisplinghoff, Cologne, Germany

## Abstract

To establish the optimal detection of third-generation cephalosporin-resistant Enterobacterales (3GCREB), the performance of four different screening methods has been investigated: stool samples without (A) and with (B) pre-enrichment and rectal swabs without (C) and with (D) pre-enrichment were contrasted. Pre-enrichment approaches (B and D) increased the detection of 3GCREB carriers by 29.4% (20/68 3GCREB carriers only found using pre-enrichment, *p* < 0.0001) compared to direct plating approaches (A and C). Moreover, the study demonstrates a minor advantage of stool samples in contrast to rectal swabs in both cases (with and without pre-enrichment). Registration number: DRKS00022520, 24 July 2020.

**Supplementary Information:**

The online version contains supplementary material available at 10.1007/s10096-021-04250-1.

The prevalence of third-generation cephalosporin-resistant Enterobacterales (3GCREB) is increasing worldwide [1]. Patients at risk often develop bacteraemia with the multidrug-resistant germs of the intestinal flora [2–4]. The initial empirical therapy of these infections is often insufficient and thereby results in an increased morbidity and mortality [5, 6]. For this purpose, many hospitals screen the intestinal flora of high-risk patients for the presence of 3GCREB before admission. Therefore, stool samples or rectal swabs are cultured on selective agar. Whereas stool samples represent the elaborated gold standard, the feasible use of rectal swabs is widely prevalent, since they can be easily obtained at any time. Lerner et al. [7] displayed a comparable performance of stool samples and rectal swabs in the detection of *Klebsiella pneumoniae* carbapenemase. Moreover, studies demonstrated that pre-enrichment has the ability to improve the detection of 3GCREB in the stool sample and also in the rectal swabs [8–10]. It is up to now unknown whether pre-enrichment of stool samples or of rectal swabs has the best sensitivity for the detection of 3GCREB. Therefore, in this study, a direct comparison of the following four methods was performed: stool samples without pre-enrichment (A), stool samples with pre-enrichment (B), rectal swabs without pre-enrichment (C) and rectal swabs with pre-enrichment (D).

From February to April 2016, 478 stool samples from 356 consecutive patients of the University Hospital Cologne submitted for 3GCREB screening were included in the study. For patients from whom there was more than one sample, the first sample was always used for the calculation on patient level and for determination of the prevalence of 3GCREB and ESBL-E carriers.

All stool samples were homogenized by vortexing for up to 1 min and then processed by one investigator within 48 h of receipt. Gender, age and ward of each patient were recorded. The following four different algorithms were compared for the detection of 3GCREB, ESBL-E and CPE (Fig. 1).

(A) *Sool sample without pre-enrichment:* A 10-μl loop full of solid stool was directly plated onto selective ChromID ESBL (bioMérieux, Marcy l’Etoile, France) and McCARB agar, a MacConkey-based agar produced in house containing ertapenem, zinc and cloxacillin [11].
(B) *Stool sample with pre-enrichment:* The whole stool sample was covered with 5 ml of a semi-selective MacConkey broth (Roth, Germany, Karlsruhe) supplemented with vancomycin (64 mg/L) and vortexed for up to 1 min. This step was performed after sampling for C and D.
(C) *Rectal swab without pre-enrichment:* A standard rayon swab in Amies medium (Copan, Brescia Italy) was used to imitate rectal swabbing. Therefore, the swab was slightly dipped into the fresh stool sample and wiped off carefully on the edge of the vial. For simulation of rectal swabbing, care was taken to handle all swabs in the same manner to assure that the amount of stool for each swab was similar and adequate. The swab was then streaked out onto ChromID ESBL and McCARB agar.
(D) *Rectal swab with pre-enrichment:* Rectal swab was prepared as described above (C). Instead of plating the swab directly on the agar, the swab was put into above-mentioned enrichment broth.

**Fig. 1:**
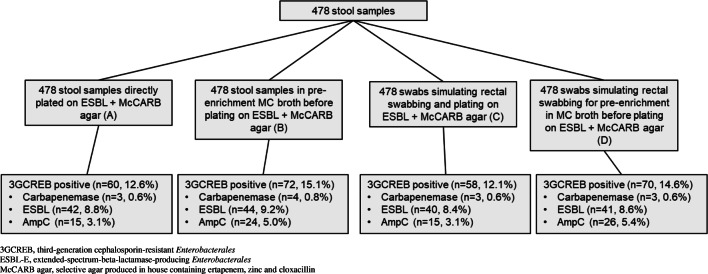
Flow chart study design and description of sample flow

Agar plates and enriched samples were incubated at 36 °C ± 1 °C in ambient air. After incubation for 18–24 h, ChromID ESBL and McCARB plates were read. Enriched samples (B/D) were vortexed for 1 min and 10 μl each subcultured onto ChromID ESBL and McCARB agar. Plates were incubated at 36 °C ± 1 °C in ambient air and read after 18–24 h. Phenotypic detection and characterisation of 3GCREB as well as molecular characterisation of the isolates were performed as previously described [9]. McCARB agar was used in addition to ESBL agar to detect Enterobacterales harbouring carbapenemases, especially OXA-48 which often show low minimum inhibitory concentration (MIC) to third-generation cephalosporins and potentially do not grow on ESBL agar as previously demonstrated [11]. A sample was considered 3GCREB/ESBL-positive, when at least one 3GCREB/ESBL-positive isolate was recovered in any of the four used algorithms. A combined gold standard was applied, consisting of all 3GCREB/ESBL-E recovered in any of the four approaches. If an isolate harboured more than one resistance mechanism, it was classified only once in the highest resistance mechanism (carbapenemase over ESBL over AmpC over SHV-1 β-lactamases/K1-hyperproducing isolates). Algorithms were compared using McNemar test.

In this study, 478 stool samples from 356 consecutive patients were analysed. Among all patients, 19.1% (68/356) were identified as positive for 3GCREB with at least one of the algorithms used, including 9.6% (34/356) as positive for ESBL-E. The prevalence of CPE carriers was 1.1% (4/356). In comparison to direct plating, the use of pre-enrichment increased the detection of 3GCREB carriers in both cases (stool samples and rectal swabs) by 17.6% (12/68, *p* = 0.004). For the detection of ESBL-E carriers, the pre-enrichment in rectal swabs and in stool samples showed an insignificant advantage (stool samples: 5.9% only found using pre-enrichment (2/34, *p* = 0.625); rectal swabs: 8.8% only found using pre-enrichment (3/34, *p* = 0.25)). Comparing approaches using stool (A and B) versus approaches with rectal swabs (C and D) for 3GCREB and ESBL-E carrier detection, there is a non-significant trend towards better detection using stool samples (3GCREB: 2.9% (2/68 only found using stool samples, *p* = 0.83); ESBL-E: 5.9% (2/34 only found using stool samples, *p* = 0.69)). The most sensitive single approach for detection of 3GCREB and ESBL-E carriers was approach B (stool samples with pre-enrichment), which demonstrated a sensitivity for 3GCREB of 82.4% (56/68) and for ESBL-E of 91.2% (31/34). Taking together pre-enrichment approaches (B and D) increased the detection of 3GCREB carriers by 29.4% (20/68, *p* < 0.0001) compared to direct plating approaches (A and C). By combining the screening methods (direct plating without pre-enrichment and plating after pre-enrichment), the detection of 3GCREB is slightly better compared to only using the pre-enrichment approach (*p* > 0.05). Where applicable, the agreements of the different approaches were compared (Table 1).

**Table 1:** Comparisons of the different algorithms for the detection of 3GCREB carriers by using McNemar

	Stool without pre-enrichment (A)	Stool with pre-enrichment (B)	Rectal swab without pre-enrichment (C)	Rectal swab with pre-enrichment (D)	Stool total (A + B)	Rectal swab total (C + D)
Stool without pre-enrichment (A)^a^	X	X	98.3/0.6875	X	X	X
Stool with pre-enrichment (B)^a^	95.5/0.0042	X	94.9/0.0013	93.8/0.8318	X	93.8/1
Rectal swab without pre-enrichment (C)^a^	X	X	X	X	X	X
Rectal swab with pre-enrichment (D)^a^	95.5/0.0213	X	95.5/0.0042	X	X	X
Stool total (A + B)^a^	96.1/0.0001	99.4/0.5	94.9/0.0001	93.8/0.5235	X	93.8/0,8318
Rectal swab total (C + D)^a^	95.5/0.0042	93.8/1	96.1/0.0001	99.4/0.5	X	X

The approach with the better performance is in each comparison located in the slot on the left side

^a^Overall percent agreement (%)/*p* value

Sensitivity, specificity, PPV and NPV for the detection of 3GCREB and ESBL-E were calculated for each algorithm on sample level. Results are shown in Table 2.

**Table 2:** Sensitivities, specificities, PPVs and NPVs of the four different algorithms for the detection of 3GCREB tested on 478 clinical specimens

Detection target (3GCREB or ESBL-E) Samples considered and used detection algorithm	No. of samples			Sensitivity, % (95% CI)	Specificity, % (95% CI)	PPV, % (95% CI)	NPV, % (95% CI)	Prevalence, % (95% CI)
	TP^a^	FP^b^	FN^c^					
Detection of 3GCREB								
All growth considered^d^								
Stool without pre-enrichment (A)	60	46	27	69.0 (58.0–78.2)	88.2 (84.5–91.2)	56.6 (46.6–66.1)	92.7 (89.5–95.1)	18.2 (14.9–22.0)
Stool with pre-enrichment (B)	72	52	15	82.8 (72.8–89.7)	86.7 (82.8–89.8)	58.1 (48.9–66.8)	95.8 (93.0–97.5)	18.2 (14.9–22.0)
Rectal swab without pre-enrichment (C)	58	29	29	66.7 (55.7–76.2)	92.6 (89.4–94.9)	66.7 (55.7–76.2)	92.6 (89.4–94.9)	18.2 (14.9–22.0)
Rectal swab with pre-enrichment (D)	70	50	17	80.5 (70.3–87.9)	87.2 (83.4–90.3)	58.3 (49.0–67.2)	95.3 (92.4–97.1)	18.2 (14.9–22.0)
Stool total (A + B)	75	64	12	86.2 (76.8–92.4)	83.6 (79.5–87.1)	54.0 (45.3. - 62.4)	96.5 (93.7–98.1)	18.2 (14.9–22.0)
Rectal swab total (C + D)	74	52	13	85.1 (75.4–91.5)	86.7 (82.8–89.8)	58.7 (49.6–67.3)	96.3 (93.6–97.9)	18.2 (14.9–22.0)
Selected growth considered^e^								
Stool without pre-enrichment (A)	60	25	27	69.0 (58.0–78.2)	93.6 (90.6–95.7)	70.6 (59.6–79.7)	93.1 (90.0–95.3)	18.2 (14.9–22.0)
Stool with pre-enrichment (B)	72	20	15	82.8 (72.8–89.7)	94.9 (92.1–96.8)	78.3 (68.2–85.9)	96.1 (93.5–97.7)	18.2 (14.9–22.0)
Rectal swab without pre-enrichment (C)	58	15	29	66.7 (55.7–76.2)	96.2 (93.6–97.8)	79.5 (68.1–87.7)	92.8 (89.8–95.1)	18.2 (14.9–22.0)
Rectal swab with pre-enrichment (D)	70	13	17	80.5 (70.3–87.9)	96.7 (94.2–98.1)	84.3 (74.3–91.1)	95.7 (93.1–97.4)	18.2 (14.9–22.0)
Stool total (A + B)	75	29	12	86.2 (76.8–92.4)	92.6 (89.4–94.9)	72.1 (62.3–80.2)	96.8 (94.3–98.3)	18.2 (14.9–22.0)
Rectal swab total (C + D)	74	17	13	85.1 (75.4–91.5)	95.7 (93.0–97.4)	81.3 (71.5–88.4)	96.6 (94.2–98.1)	18.2 (14.9–22.0)
Detection of ESBL-E								
All growth considered^d^								
Stool without pre-enrichment (A)	42	64	6	87.5 (74.1–94.8)	85.1 (81.3–88.3)	39.6 (30.4–49.6)	98.4 (96.3–99.3)	10.0 (7.6–13.2)
Stool with pre-enrichment (B)	44	80	4	91.7 (79.1–97.3)	81.4 (77.3–84.9)	35.5 (27.2–44.6)	98.9 (96.9–99.6)	10.0 (7.6–13.2)
Rectal swab without pre-enrichment (C)	40	47	8	83.3 (69.2–92.0)	89.1 (85.6–91.8)	46.0 (35.4–57.0)	98.0 (95.8–99.0)	10.0 (7.6–13.2)
Rectal swab with pre-enrichment (D)	41	79	7	85.4 (71.6–93.5)	81.6 (77.6–85.1)	34.2 (25.9–43.5)	98.0 (95.8–99.1)	10.0 (7.6–13.2)
Stool total (A + B)	46	93	2	95.8 (84.6–99.3)	78.4 (74.1–82.1)	33.1 (25.5–41.6)	99.4 (97.7–99.9)	10.0 (7.6–13.2)
Rectal swab total (C + D)	43	83	5	89.6 (76.6–96.1)	80.7 (76.6–84.3)	34.1 (26.1–43.2)	98.6 (96.5–99.5)	10.0 (7.6–13.2)
Selected growth considered^e^								
Stool without pre-enrichment (A)	42	43	6	87.5 (74.1–94.8)	90.0 (86.7–92.6)	49.4 (38.5–60.4)	98.5 (96.5–99.4)	10.0 (7.6–13.2)
Stool with pre-enrichment (B)	44	48	4	91.7 (79.1–97.3)	88.8 (85.4–91.6)	47.8 (37.4–58.4)	99.0 (97.2–99.7)	10.0 (7.6–13.2)
Rectal swab without pre-enrichment (C)	40	33	8	83.3 (69.2–92.0)	92.3 (89.3–94.6)	54.8 (42.8–66.3)	98.0 (96.0–99.1)	10.0 (7.6–13.2)
Rectal swab with pre-enrichment (D)	41	42	7	85.4 (71.6–93.5)	90.2 (86.9–92.8)	49.4 (38.3–60.5)	98.2 (96.2–99.2)	10.0 (7.6–13.2)
Stool total (A + B)	46	58	2	95.8 (84.6–99.3)	86.5 (82.8–89.5)	44.2 (34.6–54.3)	99.5 (97.9–99.9)	10.0 (7.6–13.2)
Rectal swab total (C + D)	43	48	5	89.6 (76.6–96.1)	88.8 (85.4–91.6)	47.3 (36.8–57.9)	98.7 (96.8–99.5)	10.0 (7.6–13.2)

A combined gold standard consisting of all four approaches was applied for the calculation

^a^TP, true positive. A result was considered TP when at least one 3GCREB isolate was recovered from the sample

^b^FP, false positive. A result was considered FP when the isolate(s) recovered from the sample was not confirmed to be a 3GCREB

^c^FN, false negative

^d^When no selection criteria were applied (for calculation, all oxidase positive/negative and all colored/uncolored colonies growing on ESBL agar were counted)

^e^When selection criteria were applied (only oxidase negative and colored colonies growing on ESBL agar were counted)

Overall, 97 3GCREB isolates were recovered from 87 positive stool samples. *Escherichia coli* was the most common species: 46.4% (45/97) of all 3GCREB. ESBL production was the most frequent resistance mechanism among all 3GCREB (54/97, 55.7%). Contrasting the pre-enrichment approaches (B and D) to direct plating (A and C), pre-enrichment increased the detection of *Citrobacter spp.* (by 250%, 25/10, *p* < 0.0001) as well as the detection of AmpC-beta-lactamases (by 219%, 35/16, *p* < 0.0001) (Table 3).

**Table 3:** Isolate characteristics of 3GCREB isolates recovered by all 4 algorithms

Strain characteristics	Overall (*n* = 97)	3GCREB isolates recovered by					
		Stool without pre-enrichment (A) (*n* = 68)	Stool with pre-enrichment (B) (*n* = 81)	Rectal swab without pre-enrichment (C) (*n* = 63)	Rectal swab with pre-enrichment (D) (*n* = 75)	Without pre-enrichment total (A + C) (*n* = 71)	With pre-enrichment total (B + D) (*n* = 94)
Species 3GCREB							
*Escherichia coli*	45 (46.4%)	42 (61.8%)	41 (50.6%)	37 (58.7%)	37 (49.3%)	42 (59.2%)	43 (45.7%)
*Klebsiella species*	11 (11.3%)	6 (8.8%)	11 (13.6%)	5 (8.0%)	7 (9.3%)	7 (9.9%)	11 (11.7%)
*Enterobacter species*	12 (12.4%)	8 (11.8%)	11 (13.6%)	9 (14.3%)	11 (14.7%)	9 (12.7%)	12 (12.8%)
*Citrobacter species*	25 (25.8%)	10 (14.7%)	15 (18.5%)	9 (14.3%)	18 (24.0%)	10 (14.1%)	25 (26.6%)
*Others*	4 (4.1%)	2 (2.9%)	3 (3.7%)	3 (4.8%)	2 (2.7%)	3 (4.2%)	3 (3.2%)
Resistance mechanism							
ESBL (no. (%))	54 (55.7%)	47 (69.1%)	49 (60.5%)	43 (68.3%)	44 (58.7%)	49 (69.0%)	52 (55.3%)
CTX-M-1 (no. (% of ESBL))	33 (61.1%)	30 (63.8%)	31 (63.3%)	29 (67.4%)	28 (63.6%)	32 (65.3%)	32 (61.5%)
CTX-M-9 (no. (% of ESBL))	9 (16.7%)	7 (14.9%)	8 (16.3%)	6 (14.0%)	8 (18.2%)	7 (14.3%)	9 (17.3%)
Unknown ESBL mechanism	12 (22.2%)	10 (21.3%)	10 (20.4%)	8 (18.6%)	8 (18.2%)	10 (20.4%)	11 (21.2%)
AmpC (no. (%))	36 (37.1%)	15 (22.1%)	25 (30.9%)	15 (23.8%)	26 (34.7%)	16 (22.5%)	35 (37.2%)
Hyper K1 (no. (%))	1 (1.0%)	1 (1.5%)	1 (1.2%)	1 (1.6%)	1 (1.3%)	1 (1.4%)	1 (1.1%)
VIM carbapenemase (no. (%))	2 (2.1%)	1 (1.5%)	2 (2.5%)	1 (1.6%)	1 (1.3%)	1 (1.4%)	2 (2.1%)
IMP carbapenemase (no. (%))	2 (2.1%)	2 (2.9%)	2 (2.5%)	2 (3.2%)	2 (2.7%)	2 (2.8%)	2 (2.1%)
SHV-1 (no. (%))	2 (2.1%)	2 (2.9%)	2 (2.5%)	1 (1.6%)	1 (1.3%)	2 (2.8%)	2 (2.1%)

3GCREB, third-generation cephalosporin-resistant *Enterobacterales*

ESBL-E, extended-spectrum-beta-lactamase-producing *Enterobacterales*

If an Isolate harboured more than one resistance mechanism, it was classified in the highest resistance mechanism (carbapenemase > ESBL > AmpC > Hyper K1/SHV-1)

However, the detection of other species, resistance mechanisms and drug susceptibilities stay almost unaffected and no substantial differences between the four approaches were found (Online Resource 1–3).

The patient characteristics of 3GCREB and ESBL-E carriers that were detected by each of the four approaches were compared to each other, but no substantial differences between the groups were found (Online Resource 4 and Online Resource 5).

Due to the rising prevalence of 3GCREB worldwide, screening measures for multidrug-resistant Enterobacterales in microbiology laboratories are increasingly important. While stool samples are considered the gold standard in 3GCREB screening, rectal swabs predominate practically as they are time-independent from defecation and there is evidence that pre-enrichment contributes to improvement. In the present study, for the first time, the performance of rectal swabs and stool swabs with and without pre-enrichment in one experimental study were compared. It could be shown that conducting pre-enrichment of stool samples and rectal swabs increased the detection of 3GCREB carriers significantly. The number of carriers that could only be found using pre-enrichment was as high as 29.4% (20/68 only found using pre-enrichment). The study results are consistent with previously published data showing that pre-enrichment improved the screening of 3GCREB from stool samples [10], nylon-flocked swabs [8] and rayon swabs [9]. Furthermore, there are no significant differences between stool samples and rectal swabs according to the amount of detected 3GCREB and ESBL-E carriers, but a minor trend was found towards the stool samples. Thus, stool samples with pre-enrichment have the highest sensitivity in the detection of 3GCREB and ESBL-E carriers. If the pre-enrichment procedure is combined with direct plating of stools on screening culture media, the highest sensitivity can be achieved. This can also compensate the prolonged turnaround time caused by pre-enrichment but results in higher costs. The study design conforms to a model of perfect swabbing, whereas under routine conditions, approximately 20% of rectal swabs are of minor quality [9]. Consequently, rectal swabs are likely favoured in comparison to the clinical situation. Using the McCARB agar did not lead to an additional detection of carbapenemase isolates, as all CPE in this study were also found on ESBL agar. However, there were no OXA-48 CPE in this study, which might have been missed by the ESBL agar [11].

Considering the data from the present study and from previous studies, we believe that screening for 3GCREB should always be performed with pre-enrichment. For optimal screening, stool samples should be favoured.

## Supplementary information

ESM 1 (PDF 276 kb)

## Declarations

All procedures performed in this study were in accordance with the ethical standards of the institutional and national research committee and with the 1964 Helsinki declaration and its later amendments or comparable ethical standards.
