# Supplementary material for: Comparison of stool samples and rectal swabs with and without pre-enrichment for the detection of third-generation cephalosporin-resistant Enterobacterales (3GCREB)
Source: Eur J Clin Microbiol Infect Dis. 2021 Apr 27;40(11):2431–6. doi: 10.1007/s10096-021-04250-1 (PMC8520522; doi:10.1007/s10096-021-04250-1)
Supplement: Supplementary file 1 — (PDF 276 kb) [file 10096_2021_4250_MOESM1_ESM.pdf]

**Comparison of stool samples and rectal swabs with and without pre-enrichment for the detection of third-generation cephalosporin-resistant *Enterobacterales* (3GCREB)**
**European Journal of Clinical Microbiology & Infectious Diseases**

Tarek Jazmati[a,b], Axel Hamprecht[a,b,d], Nathalie Jazmati[a,b,c,#]
[a]Institute for Medical Microbiology, Immunology and Hygiene, University of Cologne, Cologne, Germany; [b]German Centre for Infection Research (DZIF), partner site Bonn-Cologne, Germany; [c]Laboratory Dr. Wisplinghoff, Cologne, Germany; [d]Institute for Medical Microbiology and Virology, University of Oldenburg & Klinikum Oldenburg, Oldenburg, Germany
[#]Corresponding author: nathalie.jazmati@uk-koeln.de

**Online Resource 1** Drug susceptibilities of 3GCREB isolates recovered by the four approaches and compared to each other

| Strain characteristics | Overall (n=97) | | | 3GCREB isolates recovered by | | | | | | | | | | | |
|---|---|---|---|---|---|---|---|---|---|---|---|---|---|---|---|
| | | | | Stool without pre-enrichment (A) (n=68) | | | Stool with pre-enrichment (B) (n=81) | | | Rectal swab without pre-enrichment (C) (n=63) | | | Rectal swab with pre-enrichment (D) (n=75) | | |
| **Drug Susceptibility[a]** | **MIC$_{50}$[a]** | **MIC$_{90}$[a]** | **I/R[a] (n, %)** | **MIC$_{50}$[a]** | **MIC$_{90}$[a]** | **I/R[a] (n, %)** | **MIC$_{50}$[a]** | **MIC$_{90}$[a]** | **I/R[a] (n, %)** | **MIC$_{50}$[a]** | **MIC$_{90}$[a]** | **I/R[a] (n, %)** | **MIC$_{50}$[a]** | **MIC$_{90}$[a]** | **I/R[a] (n, %)** |
| Piperacillin/Tazobactam | 64 | >128 | 60 (61.9%) | 16 | >128 | 36 (52.9%) | 32 | >128 | 46 (56.8%) | 16 | >128 | 33 (52.4%) | 64 | >128 | 46 (61.3%) |
| Cefpodoxime | >8 | >8 | 97 (100%) | >8 | >8 | 68 (100%) | >8 | >8 | 81 (100%) | >8 | >8 | 63 (100%) | >8 | >8 | 75 (100%) |
| Cefotaxime | >64 | >64 | 92 (94.8%) | >64 | >64 | 66 (97.1%) | >64 | >64 | 77 (95.1%) | >64 | >64 | 62 (98.4%) | >64 | >64 | 73 (97.3%) |
| Ceftazidime | 16 | >64 | 76 (78.4%) | 16 | >64 | 53 (77.9%) | 16 | >64 | 65 (80.2%) | 16 | >64 | 49 (77.8%) | 16 | >64 | 59 (78.7%) |
| Ertapenem | <0.5 | 2 | 15 (15.5%) | <0.5 | 1 | 9 (13.2%) | <0.5 | 2 | 14 (17.3%) | <0.5 | 1 | 10 (15.9%) | <0.5 | 1 | 11 (14.7%) |
| Imipenem | <0.25 | 1 | 4 (4.1%) | <0.25 | 1 | 3 (4.4%) | <0.25 | 1 | 4 (4.9%) | <0.25 | 0.5 | 3 (4.8%) | <0.25 | 1 | 3 (4.0%) |
| Meropenem | <0.25 | <0.25 | 2 (2.1%) | <0.25 | <0.25 | 1 (1.5%) | <0.25 | <0.25 | 2 (2.5%) | <0.25 | <0.25 | 1 (1.6%) | <0.25 | <0.25 | 1 (1.3%) |
| Ciprofloxacine | <0.25 | >4 | 38 (39.2%) | <0.25 | >4 | 30 (44.1%) | <0.25 | >4 | 33 (40.7%) | <0.25 | >4 | 25 (39.7%) | <0.25 | >4 | 29 (38.7%) |
| Trimethoprim/Sulfamethoxazole | <20 | >320 | 43 (44.3%) | >320 | >320 | 35 (51.5%) | <20 | >320 | 38 (46.9%) | <20 | >320 | 30 (47.6%) | <20 | >320 | 34 (45.3%) |

[a] MIC$_{50}$/MIC$_{90}$ estimates the antibiotic concentration (mg/liter) that inhibits 50% (MIC$_{50}$) and 90% (MIC$_{90}$) of tested bacterial isolates; I/R, number of isolates considered to be intermediate (I) or resistant (R) to the indicated antimicrobial agent according to EUCAST clinical MIC breakpoints.

[*] p-value was obtained using χ2 test and accounts for the comparison of 3GCREB isolates recovered by the four approaches.

3GCREB, third-generation cephalosporin-resistant *Enterobacterales*

**Comparison of stool samples and rectal swabs with and without pre-enrichment for the detection of third-generation cephalosporin-resistant *Enterobacterales* (3GCREB)**
**European Journal of Clinical Microbiology & Infectious Diseases**

Tarek Jazmati[a,b], Axel Hamprecht[a,b,d], Nathalie Jazmati[a,b,c,#]
[a]Institute for Medical Microbiology, Immunology and Hygiene, University of Cologne, Cologne, Germany; [b]German Centre for Infection Research (DZIF), partner site Bonn-Cologne, Germany; [c]Laboratory Dr. Wisplinghoff, Cologne, Germany; [d]Institute for Medical Microbiology and Virology, University of Oldenburg & Klinikum Oldenburg, Oldenburg, Germany
[#]Corresponding author: nathalie.jazmati@uk-koeln.de

**Online Resource 2** Strain characteristics of ESBL-E isolates recovered by the four approaches and compared to each other

| Strain characteristics | Overall (n=54) | ESBL-E isolates recovered by | | | |
|---|---|---|---|---|---|
| | | Stool without pre-enrichment (A) (n=47) | Stool with pre-enrichment (B) (n=49) | Rectal swab without pre-enrichment (C) (n=43) | Rectal swab with pre-enrichment (D) (n=44) |
| **Species** | | | | | |
| *Escherichia coli* | 45 (83.3%) | 42 (89.4%) | 41 (83.7%) | 37 (86.0%) | 37 (84.1%) |
| *Klebsiella pneumoniae* | 3 (5.6%) | 3 (6.4%) | 3 (6.1%) | 2 (4.7%) | 2 (4.5%) |
| *Klebsiella aerogenes* | 2 (3.7%) | 0 | 2 (4.1%) | 1 (2.3%) | 1 (2.3%) |
| *Enterobacter cloacae* | 3 (5.6%) | 2 (4.3%) | 3 (6.1%) | 3 (7.0%) | 3 (6.8%) |
| *Citrobacter farmeri* | 1 (1.9%) | 0 | 0 | 0 | 1 (2.3%) |
| **ESBL genes** | | | | | |
| CTX-M-1 (no. (% of ESBL)) | 33 (61.1%) | 30 (63.8%) | 31 (63.3%) | 29 (67.4%) | 28 (63.6%) |
| CTX-M-9 (no. (% of ESBL)) | 9 (16.7%) | 7 (14.9%) | 8 (16.3%) | 6 (14.0%) | 8 (18.2%) |
| Unknown (no. (% of ESBL)) | 12 (22.2%) | 10 (21.3%) | 10 (20.4%) | 8 (18.6%) | 8 (18.2%) |

| **Drug Susceptibilities[a]** | Overall MIC50 | Overall MIC90 | Overall I/R (n, %) | A MIC50 | A MIC90 | A I/R (n, %) | B MIC50 | B MIC90 | B I/R (n, %) | C MIC50 | C MIC90 | C I/R (n, %) | D MIC50 | D MIC90 | D I/R (n, %) |
|---|---|---|---|---|---|---|---|---|---|---|---|---|---|---|---|
| Piperacillin/Tazobactam | 8 | >128 | 19 (35.2%) | 8 | >128 | 16 (34.0%) | 8 | >128 | 16 (32.7%) | 8 | >128 | 14 (32.6%) | 8 | >128 | 16 (36.4%) |
| Cefpodoxime | >8 | >8 | 54 (100%) | >8 | >8 | 47 (100%) | >8 | >8 | 49 (100%) | >8 | >8 | 43 (100%) | >8 | >8 | 44 (100%) |
| Cefotaxime | >64 | >64 | 53 (98.1%) | >64 | >64 | 46 (97.9%) | >64 | >64 | 48 (98.0%) | >64 | >64 | 42 (97.7%) | >64 | >64 | 44 (100%) |
| Ceftazidime | 8 | >64 | 37 (68.5%) | 8 | >64 | 32 (68.1%) | 8 | >64 | 35 (71.4%) | 8 | >64 | 30 (69.8%) | 8 | >64 | 30 (68.2%) |
| Ertapenem | <0.5 | <0.5 | 2 (3.7%) | <0.5 | <0.5 | 2 (4.3%) | <0.5 | <0.5 | 2 (4.1%) | <0.5 | <0.5 | 2 (4.7%) | <0.5 | <0.5 | 2 (4.5%) |
| Imipenem | <0.25 | <0.25 | 0 | <0.25 | <0.25 | 0 | <0.25 | <0.25 | 0 | <0.25 | 0.5 | 0 | <0.25 | 0.5 | 0 |
| Meropenem | <0.25 | <0.25 | 0 | <0.25 | <0.25 | 0 | <0.25 | <0.25 | 0 | <0.25 | <0.25 | 0 | <0.25 | <0.25 | 0 |
| Ciprofloxacine | >4 | >4 | 32 (59.3%) | >4 | >4 | 28 (59.6%) | >4 | >4 | 28 (57.1%) | 2 | >4 | 23 (53.5%) | >4 | >4 | 26 (59.1%) |
| Trimethoprim/Sulfamethoxazole | >320 | >320 | 37 (68.5%) | >320 | >320 | 33 (70.2%) | >320 | >320 | 33 (67.3%) | >320 | >320 | 29 (67.4%) | >320 | >320 | 32 (72.7%) |

[a] $MIC_{50}$/$MIC_{90}$ estimates the antibiotic concentration (mg/l) that inhibits 50% ($MIC_{50}$) and 90% ($MIC_{90}$) of tested bacterial isolates; I/R, number of isolates considered to be intermediate (I) or resistant (R) to the indicated antimicrobial agent according to EUCAST clinical MIC breakpoints.

ESBL-E, extended-spectrum-beta-lactamase-producing *Enterobacterales*

**Comparison of stool samples and rectal swabs with and without pre-enrichment for the detection of third-generation cephalosporin-resistant *Enterobacterales* (3GCREB)**
**European Journal of Clinical Microbiology & Infectious Diseases**

Tarek Jazmati[a,b], Axel Hamprecht[a,b,d], Nathalie Jazmati[a,b,c,#]
[a]Institute for Medical Microbiology, Immunology and Hygiene, University of Cologne, Cologne, Germany; [b]German Centre for Infection Research (DZIF), partner site Bonn-Cologne, Germany; [c]Laboratory Dr. Wisplinghoff, Cologne, Germany; [d]Institute for Medical Microbiology and Virology, University of Oldenburg & Klinikum Oldenburg, Oldenburg, Germany
[#]Corresponding author: nathalie.jazmati@uk-koeln.de

**Online Resource 3** Distribution of identified species and resistance mechanisms among the 97 3GCREB study isolates

| | ESBL (n (%)) | | | | Other resistance mechanism (n(%)) | | | | | Total (n (%))[4] |
|---|---|---|---|---|---|---|---|---|---|---|
| | CTX-M-1 group [2] | CTX-M-9 group [2] | unknwon ESBL[2] | **Total ESBL[1]** | **AmpC [1]** | **Hyper K1 [1]** | ***bla*$_{IMP}$ [1]** | ***bla*$_{VIM}$ [1]** | **SHV [1]** | |
| *Escherichia coli* | 29 (64.4) | 7 (15.6) | 9 (20.0) | 45 (100) | - | - | - | - | - | 45 (46.4) |
| *Klebsiella pneumoniae* | 2 (66.7) | - | 1 (33.3) | 3 (50.0) | 1 (16.7) | - | - | - | 2 (33.3) | 6 (6.2) |
| *Klebsiella oxytoca* | - | - | - | - | - | 1 (100) | - | - | - | 1 (1.0) |
| *Klebsiella aerogenes* | 1 (50.0) | - | 1 (50.0) | 2 (50.0) | 2 (50.0) | - | - | - | - | 4 (4.1) |
| *Enterobacter cloacae* | 1 (33.3) | 2 (66.7) | - | 3 (37.5) | 5 (62.5) | - | - | - | - | 8 (8.2) |
| *Other Enterobacter spp.* | - | - | - | - | 4 (100) | - | - | - | - | 4 (4.1) |
| *Citrobacter freundii* | - | - | - | - | 17 (89.5) | - | - | 2 (10.5) | - | 19 (19.6) |
| *Citrobacter braakii* | - | - | - | - | 4 (100) | - | - | - | - | 4 (4.1) |
| *Other Citrobacter spp.* | - | - | 1 (100) | 1 (50.0) | 1 (50.0) | - | - | - | - | 2 (2.1) |
| *Hafnia alvei* | - | - | - | - | 2 (100) | - | - | - | - | 2 (2.1) |
| *Serratia marcescens* | - | - | - | - | - | - | 2 (100) | - | - | 2 (2.1) |
| **Total (n (%))** | 33 (61.1)[3] | 9 (16.7) [3] | 12 (22.2) [3] | **54 (55.7) [4]** | **36 (37.1)[4]** | **1 (1.0) [4]** | **2 (2.1) [4]** | **2 (2.1) [4]** | **2 (2.1) [4]** | **97 (100)** |

[1]Percentage based on all isolates of the named species

[2]Percentage based on total number of ESBL positive isolates of the named species

[3]Percentage based on total number of ESBL positive isolates

[4]Percentage based on all 97 isolated 3GCREB

3GCREB, third-generation cephalosporin-resistant *Enterobacterales*; ESBL-E, extended-spectrum-beta-lactamase-producing *Enterobacterales*

**Comparison of stool samples and rectal swabs with and without pre-enrichment for the detection of third-generation cephalosporin-resistant *Enterobacterales* (3GCREB)**
**European Journal of Clinical Microbiology & Infectious Diseases**

Tarek Jazmati[a,b], Axel Hamprecht[a,b,d], Nathalie Jazmati[a,b,c,#]
[a]Institute for Medical Microbiology, Immunology and Hygiene, University of Cologne, Cologne, Germany; [b]German Centre for Infection Research (DZIF), partner site Bonn-Cologne, Germany; [c]Laboratory Dr. Wisplinghoff, Cologne, Germany; [d]Institute for Medical Microbiology and Virology, University of Oldenburg & Klinikum Oldenburg, Oldenburg, Germany
[#]Corresponding author: nathalie.jazmati@uk-koeln.de

**Online Resource 4** Patient characteristics of 3GCREB carriers detected by the four approaches and compared to each other

| Patients characteristics[a] | Overall carrier (n=68) | 3GCREB carrier recovered by | | | |
|---|---|---|---|---|---|
| | | Stool without pre-enrichment (A) (n=44) | Stool with pre-enrichment (B) (n=56) | Rectal swab without pre-enrichment (C) (n=42) | Rectal swab with pre-enrichment (D) (n=54) |
| Age (years) | 58.9 ± 22.2 | 59.1 ± 22.8 | 59.8 ± 21.5 | 56.1 ± 24.4 | 56.8 ± 23.4 |
| No. Male (%) | 40 (58.8%) | 28 (63.6%) | 33 (58.9%) | 26 (61.9%) | 31 (57.4%) |
| Clinical setting (no. (%)) | | | | | |
| Internal Medicine | 33 (48.5%) | 21 (47.7%) | 27 (48.2%) | 20 (47.6%) | 28 (51.9%) |
| Surgery | 8 (11.8%) | 4 (9.1%) | 7 (12.5%) | 3 (7.1%) | 5 (9.3%) |
| Intensive Care Unit | 13 (19.1%) | 10 (22.7%) | 12 (21.4%) | 8 (19.0%) | 10 (18.5%) |
| Pediatrics | 5 (7.4%) | 3 (6.8%) | 3 (5.4%) | 4 (9.5%) | 5 (9.3%) |
| Others | 9 (13.2%) | 6 (13.6%) | 7 (12.5%) | 7 (16.7%) | 6 (11.1%) |

[a] Calculation is based on a patients level (i.e., patients with more than one positive sample were only included in the calculation once).

* p-value by $\chi 2$ test was calculated for 3GCREB carriers detected by the four approaches

3GCREB, third-generation cephalosporin-resistant *Enterobacterales*

**Comparison of stool samples and rectal swabs with and without pre-enrichment for the detection of third-generation cephalosporin-resistant *Enterobacterales* (3GCREB)**
**European Journal of Clinical Microbiology & Infectious Diseases**

Tarek Jazmati[a,b], Axel Hamprecht[a,b,d], Nathalie Jazmati[a,b,c,#]
[a]Institute for Medical Microbiology, Immunology and Hygiene, University of Cologne, Cologne, Germany; [b]German Centre for Infection Research (DZIF), partner site Bonn-Cologne, Germany; [c]Laboratory Dr. Wisplinghoff, Cologne, Germany; [d]Institute for Medical Microbiology and Virology, University of Oldenburg & Klinikum Oldenburg, Oldenburg, Germany
[#]Corresponding author: nathalie.jazmati@uk-koeln.de

**Online Resource 5** Patient characteristics of ESBL-E carriers detected by the four approaches and compared to each other

| Patients characteristics[a] | Overall carrier (n=34) | ESBL-E carrier recovered by | | | |
|---|---|---|---|---|---|
| | | Stool without pre-enrichment (A) (n=29) | Stool with pre-enrichment (B) (n=31) | Rectal swab without pre-enrichment (C) (n=27) | Rectal swab with pre-enrichment (D) (n=30) |
| Age (years) | 59.2 ± 20.1 | 58.9 ± 17.4 | 58.1 ± 20.7 | 54.8 ± 20.1 | 56.6 ± 19.9 |
| No. Male (%) | 20 (58.8%) | 20 (69.0%) | 19 (61.3%) | 18 (66.7%) | 18 (60.0%) |
| Clinical setting (no. (%)) | | | | | |
| Internal Medicine | 21 (61.8%) | 18 (62.1%) | 19 (61.3%) | 18 (66.7%) | 20 (66.7%) |
| Surgery | 3 (8.8%) | 2 (6.9%) | 3 (9.7%) | 1 (3.7%) | 1 (3.3%) |
| Intensive Care Unit | 6 (17.6%) | 6 (20.7%) | 5 (16.1%) | 4 (14.8%) | 5 (16.7%) |
| Pediatrics | 1 (2.9%) | 0 | 1 (3.2%) | 1 (3.7%) | 1 (3.3%) |
| Others | 3 (8.8%) | 3 (10.3%) | 3 (9.7%) | 3 (11.1%) | 3 (10.0%) |

[a] Calculation is based on a patients level (i.e., patients with more than one positive sample were only included in the calculation once).

* p-value by $\chi 2$ test was calculated for ESBL-E carriers detected by the four approaches

ESBL-E, extended-spectrum-beta-lactamase-producing *Enterobacterales*
